# PW03-025 - Procaspase-1 contributes to inflammation via NF-KB

**DOI:** 10.1186/1546-0096-11-S1-A251

**Published:** 2013-11-08

**Authors:** M Heymann, S Winkler, S Özen, E Yilmaz, T Kallinich, A Rösen-Wolff, J Roesler, S Hofmann

**Affiliations:** 1 Dept of Pediatrics, University Hospital Dresden, Dresden, Germany; 2 Faculty of Medicine Ankara, Hacettepe University, Ankara, Turkey; 3 Dept for Pediatric Pneumology & Immunology, Charité Medical University of Berlin, Berlin, Germany

## Introduction

Caspase-1 is a pro-inflammatory enzyme which gets activated by autoprocessing following the assembly of multiprotein complexes called inflammasomes. Mature caspase-1 is responsible for the activation of the pro-inflammatory cytokines interleukin (IL)-1β and IL-18. Luksch and colleagues reported naturally occurring *CASP1* genetic variants in patients suffering from unexplained recurrent febrile episodes. Paradoxically, in vitro and in vivo analyses revealed decreased enzymatic activity of these caspase-1 variants leading to impaired cytokine production. A study of Lamkanfi and colleagues provides a possible explanation by indicating a link between enzymatically inactive procaspase-1 and activation of NF-κB, a pro-inflammatory transcription factor.

## Objectives

We tried to solve the indicated paradox by analyzing NF-κB activation in the presence of the procaspase-1 variants found.

## Methods

NF-κB activity was determined using a luciferase reporter assay system in transfected HEK 293T cells. RIP2 cleavage and ubiquitination studies were also performed in these cells. Protein/protein interactions of RIP2 and procaspase-1 were investigated in THP-1 cells by co-immunoprecipitation and in human monocyte derived macrophages by confocal fluorescence microscopy.

## Results

Procaspase-1 variants with reduced enzymatic activity increased NF-κB activation by interacting with RIP2 (receptor interacting protein kinase 2). In contrast, wildtype (wt) procaspase-1 reduced NF-κB activity by cleaving RIP2 and decreasing RIP2 ubiquitination which is essential for NF-κB activation. In addition to transfection experiments, we showed RIP2/procaspase-1 interaction in the human monocyte cell line THP-1 and in human monocyte derived macrophages after stimulation with LPS in a time dependent manner.

## Conclusion

Our results support the hypothesis that procaspase-1 variants with reduced enzymatic activity bind to RIP2 and thereby increase NF-κB activitation. This may contribute to pro-inflammatory signalling and thereby contribute to unexplained recurrent febrile episodes in the patients.

## Disclosure of interest

None declared

